# Non-Neovascular Age-Related Macular Degeneration Assessment: Focus on Optical Coherence Tomography Biomarkers

**DOI:** 10.3390/diagnostics14070764

**Published:** 2024-04-03

**Authors:** Daniela Adriana Iliescu, Ana Cristina Ghita, Larisa Adriana Ilie, Suzana Elena Voiculescu, Aida Geamanu, Aurelian Mihai Ghita

**Affiliations:** 1 Department of Physiology, “Carol Davila” University of Medicine and Pharmacy, 8 Eroii Sanitari Bld., 050474 Bucharest, Romania; suzana.voiculescu@umfcd.ro (S.E.V.); ghita.amg@gmail.com (A.M.G.); 2 Ocularcare Ophthalmology Clinic, 128 Ion Mihalache Bld., 012244 Bucharest, Romania; eyeana.ghita@gmail.com (A.C.G.); larisaadriana1907@gmail.com (L.A.I.); 3 Ophthalmology Department, Bucharest University Emergency Hospital, 169 Independence Street, 050098 Bucharest, Romania; dr.aidageamanu@gmail.com

**Keywords:** age-related macular degeneration, OCT biomarker, geographic atrophy, iRORA, cRORA

## Abstract

The imagistic evaluation of non-neovascular age-related macular degeneration (AMD) is crucial for diagnosis, monitoring progression, and guiding management of the disease. Dry AMD, characterized primarily by the presence of drusen and retinal pigment epithelium atrophy, requires detailed visualization of the retinal structure to assess its severity and progression. Several imaging modalities are pivotal in the evaluation of non-neovascular AMD, including optical coherence tomography, fundus autofluorescence, or color fundus photography. In the context of emerging therapies for geographic atrophy, like pegcetacoplan, it is critical to establish the baseline status of the disease, monitor the development and expansion of geographic atrophy, and to evaluate the retina’s response to potential treatments in clinical trials. The present review, while initially providing a comprehensive description of the pathophysiology involved in AMD, aims to offer an overview of the imaging modalities employed in the evaluation of non-neovascular AMD. Special emphasis is placed on the assessment of progression biomarkers as discerned through optical coherence tomography. As the landscape of AMD treatment continues to evolve, advanced imaging techniques will remain at the forefront, enabling clinicians to offer the most effective and tailored treatments to their patients.

## 1. Introduction

Age-related macular degeneration (AMD) stands as a leading cause of visual impairment and irreversible blindness in the elderly population worldwide. This complex, multifactorial disease is characterized by progressive deterioration of the central retina, culminating in significant vision loss and a profound impact on quality of life. The intricate pathophysiology of AMD involves a confluence of genetic predispositions, environmental factors, and metabolic dysregulation, leading to structural and functional disruptions within the macula. Advances in imaging technology have revolutionized the diagnostic approach to AMD, enabling clinicians to visualize retinal changes with remarkable precision and correlate these findings with the underlying disease mechanisms [1,2,3,4].

The advent of non-invasive imaging modalities, such as optical coherence tomography (OCT), fundus autofluorescence (FAF), and OCT angiography (OCTA), has provided invaluable insights into the retinal and choroidal alterations associated with AMD. These imaging techniques not only facilitate the detection and characterization of hallmark features of AMD, such as drusen, geographic atrophy (GA), and neovascularization, but also aid in the monitoring of disease progression and response to therapy [5,6].

Understanding the pathophysiological changes of AMD through imaging has substantial clinical implications. It allows for a more precise staging of the disease, identification of prognostic indicators, and customization of treatment strategies. This synergy between pathophysiology and imaging evaluation represents a paradigm shift in the management of AMD, emphasizing a personalized medicine approach that targets the specific needs of each patient. This review begins by thoroughly detailing the pathophysiological mechanisms underlying AMD, especially drusen development, and proceeds to summarize the imaging techniques used in assessing non-neovascular AMD. Particular attention is given to the identification of progression biomarkers via OCT.

## 2. Pathophysiology and Risk Factors of AMD

### 2.1. Oxidative Stress in AMD Pathogenesis

Several mechanisms have been implicated in the pathogenesis of retinal drusen and emergence of AMD, attributable to oxidative stress, mitochondrial dysfunction, and dysregulated lipid and protein metabolism. Oxidative stress within the retina is exacerbated by chronic UVA exposure affecting all retinal structures. Moreover, blue light exposure is known to induce oxidative stress within the retinal pigment epithelium (RPE) layer [7,8]. The cumulative oxidative stress from UV and blue light, which is further potentiated by cigarette smoke and the aging process, elevates the reactive oxygen species (ROS) levels produced by the RPE. Additionally, the phagocytosis of the outer segments of the retina, which are rich in polyunsaturated fatty acids, contributes to oxidative stress in the RPE [9]. This activity increases with age and is further influenced by the inflammatory state, culminating in RPE degeneration [10]. Oxidative stress, marked by an overproduction of ROS and a diminished reduction capacity, induces a para-inflammatory response [10]. Oxidative alteration of fatty acids leads to the formation of carboxyethylpyrrole adducts and advanced glycation end-products, which are com ponents found within drusen. These products are ligands for the receptor of advanced glycation end products (RAGEs), triggering an inflammatory response via the nuclear factor-κB pathway [11,12].

The organism mounts a natural defense against oxidative stress through the induction of an intrinsic antioxidant response, which is orchestrated by the nuclear factor erythroid 2–related factor 2 (Nrf2) and the peroxisome proliferator-activated receptor gamma coactivator-1 alpha (PGC-1α) [11,13,14]. This defensive mechanism entails the translocation of Nrf2 from the cytosol to the nucleus and subsequent activation of autophagy and proteolysis enzyme genes. A deficiency in Nrf2 correlates with increased vulnerability to oxidative damage and protein aggregation [13,14]. The PGC-1 family targets the mitochondrial antioxidant system through transcriptional control of mitochondrial biosynthesis and respiratory function.

Finally, oxidative stress leads to the accumulation of lipofuscin within the lysosomal compartment, which impedes lysosomal degradation, thus contributing to the accumulation of protein and lipid extracellular deposits. These deposits form between the basal lamina of the RPE and the inner collagenous stratum of Bruch’s membrane [15,16].

Dietary supplementation with antioxidants and omega-3 fatty acids has been investigated in clinical trials for AMD, such as AREDS1 and AREDS2. These trials underscored the correlation between the intake of antioxidant nutrients and a reduction in the incidence of late-stage AMD. They also noted the limited effectiveness of certain supplements, including omega-3 fatty acids, lutein, and zeaxanthin [17,18].

### 2.2. Mitochondrial Dysfunction and Its Role in Macular Drusen

Mitochondrial dysfunction is another pivotal factor in the pathophysiology of drusen formation. Patients with macular drusen exhibit a reduction in ATP synthesis, evidenced by decreased expression of the α-, β-, and γ- subunits of ATP synthetase in mitochondria [19]. Such mitochondrial dysfunction, characterized by various deficiencies within the respiratory chain, leads to reduced ATP production. The role of mtHsp70 is essential for the admission into as well as folding and assembly of nuclear-encoded proteins into mitochondria, vital for beta oxidation and the Krebs cycle [19,20]. Several studies suggest a potential shortfall in the mitochondrial import of nuclear-encoded proteins, thus disrupting essential cellular functions [19]. The resultant mitochondrial dysfunction increases ROS production. The non-specific oxidative damage induced by excessive mitochondrial ROS plays an important role in the aging process and the genesis of macular drusen [21]. Additionally, poly (ADP-ribosylation) is critical for maintaining genomic integrity and RNA transcription. However, under conditions of oxidative stress and energy depletion, this protective mechanism can paradoxically contribute to mitochondrial failure and consequent RPE death [22,23].

### 2.3. Lipid and Protein Dysregulation in AMD Pathogenesis

Lipids account for more than 40 percent of drusen composition, indicating that a key mechanism in drusen formation is the impairment of lipid metabolism. Dysregulation of protein homeostasis, cholesterol, and bis-monoacylglycerol phosphate (BMP) metabolism is associated with the accumulation of lipofuscin and melanolipofuscin, underscoring a metabolic deficiency [24]. Unrestrained oxidative stress also disrupts protein homeostasis in the RPE. A consequent reduction in the antioxidant defense system, an increase in ROS levels, and insufficiency in protein clearance mechanisms lead to an accumulation of damaged proteins. These proteins contribute to mitochondrial dysfunction and the formation of toxic protein aggregates [25,26]. Such changes affect RPE activity, especially the heterophagy process involving photoreceptor (PR) outer segments, and result in significant metabolic byproducts. The presence of misfolded proteins and oxidatively modified saturated fatty acids in drusen significantly hampers intracellular transport, compromising RPE functionality [27,28]. Elevated levels of high-density lipoproteins are positively correlated with AMD and the presence of cholesterol-rich deposits beneath the RPE and in subretinal regions [29,30]. In regard to cholesterol, the excess leads to an augmentation of bis (monoacyl) glycerophosphate in RPE lysosomes. Bis (monoacyl) glycerophosphate serves as a cofactor for acid sphingomyelinase which catalyzes the hydrolysis of the sphingomyelin to ceramide. Ceramide levels are elevated in AMD patients compared to controls [31]. This elevation influences the microtubule transport and membrane dynamics contributing to the pathogenesis of the disease [31,32].

The capacity of RPE to metabolize the lipids declines with age which is, as already highlighted, a key factor for generating retinal drusen [33]. Drusen are substantially comprised of lipids from PR large lipoproteins, including both esterified and unesterified cholesterol, as well as phosphatidylcholine, with the composition varying among different subtypes [33]. Subretinal drusenoid deposits (SDDs) contain large amounts of unesterified cholesterol and lack esterified cholesterol, whereas sub-RPE drusen contain both types of cholesterol [34,35]. A distinction between these drusen types also lies in their content of proteins and minerals. SDDs contain histochemical detectable apolipoprotein E, vitronectin, complement factor H, and CD59, while sub-RPE drusen are characterized by apolipoprotein B, E-containing, cholesterol-rich lipoproteins secreted by the RPE [35,36]. The structural and compositional heterogeneity within drusen, encompassing lipids, carbohydrates, and proteins, affects their appearance in OCT imaging. Spectral-domain OCT (SD OCT) has identified transiently reflective substructures beneath the RPE, categorizing them into four phenotypic subtypes: low-reflective core, high-reflective core, split drusen, and conical debris. A typical progression pattern from low-reflective core and split drusen to high-reflective particles and eventually to conical debris is observed. The pathophysiological mechanisms underlying this progression are not fully elucidated [37,38].

### 2.4. Lipofuscin Accumulation in AMD Pathogenesis

In addition to lipid deposits in AMD, the RPE also accumulates lipofuscin, melanolipofuscin, and melatonin. The distribution of melanolipofuscin and lipofuscin varies across the macular region, with low levels of lipofuscin and a higher presence of melanolipofuscin in the fovea, whereas the perifoveal region exhibits a predominance of lipofuscin [39,40,41]. Studies suggest that the distribution of these substances may be related to the type of PR present in these regions [39]. Early stages of AMD are marked by degenerative changes in the RPE–photoreceptor complex, presenting as macular areas with variable pigmentation (hypo- or hyperpigmentations) [42,43]. Lipofuscin is considered to represent accumulations of undigested cellular materials arising from phagocytosis and autophagy processes [44,45]. Lipofuscin found in RPE cells is mainly derived from the chemically modified residues of incompletely digested PR outer segments. It is a heterogeneous material composed of a mixture of peroxidized lipids, phospholipids, and different fluorescent compounds, forming endocytoplasmic granules under both physiological conditions and pathological situations [42,46,47]. Autofluorescence of the retina primary results from the lipofuscin present within RPE granules. The increase, loss, or the redistribution of retinal autofluorescence are among the earliest signs of AMD [43,46]. The major contributors to the fluorescence of lipofuscin are bis-retinoids which are amphoteric compounds that result from the vitamin A metabolism in the PR outer segment discs and diffuse into the lysosomal granules of RPE [44,46,47]. These metabolites contain cytotoxic carbonyl groups that can modify cellular proteins and lipids causing a secondary activation of the inflammatory response. Lipofuscin can induce the expression of cytokines, interleukins, VEGF, and other inflammatory mediators. Furthermore, bis-retinoids and their photooxidation and photodegradation products, as components of lipofuscin, act as photosensitizers, exerting toxic effects on RPE and retinal cells, impacting DNA and chromosomal structure, and contributing to retinal atrophy [48,49].

### 2.5. Impact of Cytochrome P450 in AMD Pathogenesis

Another important factor for aging-related macular changes is the activity of the cytochrome P450. Several studies have described an association between the activity of cytochrome P450 and neovascular age-related macular degeneration (nAMD). The cytochrome P450 monooxygenase lipid metabolites have been identified as key players in the development of angiogenesis in AMD disease models [50,51]. Indeed, overexpression of the monooxygenase cytochrome P450, C8, or inhibition of the soluble epoxide hydrolase enzyme led to increased levels of epoxygenated fatty acids with attenuated choroidal neovascularization development [52]. Endogenous levels of cytochrome P450 metabolites are highly modulated by dietary intake of omega-3 and omega-6 polyunsaturated fatty acids, endogenous synthesis, and subsequent metabolism [53]. A reduction in cytochrome P450 activity has also been associated with elevated levels of long-chain polyunsaturated fatty acids [54].

### 2.6. Genetic Variants Related to AMD Pathogenesis

Genetic variants related to drusen have been assessed through logistic regression analysis between AMD stages and single-nucleotide polymorphisms. ATP Binding Cassette Subfamily A Member 1 (ABCA1) and lipase C (LIPC) are related to the presence of intermediate and large drusen, while complement factor H, C3, C2, and age-related maculopathy susceptibility 2/HTRA serine peptidase 1 (ARMS2/HTRA1) are associated with large drusen and advanced AMD [36,54,55,56,57,58]. Apolipoprotein E is involved in lipid metabolism and serves as an important component in the transportation of cholesterol through the organism [59]. Its association with specific types of drusen suggests a role in the pathological processes underlying AMD [58]. Variations in the apolipoprotein E (APOE) gene, specifically the APOE2 allele, are associated with an increased risk, while the APOE4 allele is associated with a reduced risk compared to the APOE3 allele. Elevated plasma levels of apolipoprotein E have been documented in these patients [36,54]. Studies reveal a predominance of soft drusen within the macula, frequently associated with globular drusen and higher apolipoprotein reactivity in the retinal periphery [54,60].

### 2.7. Extracellular Matrix Regulation in AMD Pathogenesis

The regulation of extracellular matrix (ECM) levels is governed by the equilibrium between matrix metalloproteinases (MMPs) and tissue inhibitors of metalloproteinases (TIMPs). In AMD, the turnover of ECM undergoes alterations attributed to an imbalance in MMPs/TIMPs [61,62,63,64]. The expression of MMPs under normal conditions is low and strictly regulated. Various mechanisms contribute to the overexpression of MMPs, including oxidative stress, C3 activation of the complement system, cytokines, interleukins, growth factors and hormones, prostaglandins, and polymorphism of high-temperature-required protein A1 [64,65]. MMPs are responsible for proteolytic processes in the Bruch membrane and lysis of different substrates such as elastin, gelatin, and collagen I, IV, and V [64]. ECM is modified by the upregulation of MMPs’ expression in RPE cells in the presence of a lower level of TIMPs. TGF beta 2 and platelet-derived growth factor BB increase the expression of MMPs and facilitate RPE cell migration [62,65].

### 2.8. Lysosomal and Proteolysis Dysfunctions in AMD Pathogenesis

In AMD, the alteration of the lysosomal and ubiquitin-proteasome system is present. Exposure to ROS leads to the generation of degraded proteins and oxidized lipids, initiating a proactive mechanism that impacts the RPE, generating more ROS, inflammation, and establishing a positive feedback loop. The accumulation of modified proteins exerts a proinflammatory effect and promotes angiogenesis. Under normal conditions, the autophagosomes and the ubiquitin-proteasome system maintain protein homeostasis, preventing the formation of subretinal deposits [15,66]. Aging, however, is associated with a reduced expression of genes related to the ubiquitin-proteasome system, leading to diminished proteolytic activity. Consequently, the autophagy system compensates for proteolysis [67,68,69]. Dysregulation of protein homeostasis also extends to proteins in the respiratory chain system of mitochondria. A reduction in mitochondrial activity increases ROS levels [70]. Complementary to the dysregulation of mitochondrial heat shock protein 70 (mtHsp70), sequestosome 1 protein (p62/SQSTM1) plays multiple roles in clearing degraded proteins and preventing the accumulation of ROS secondary to Nrf2 deficiency [20,25,71]. The role of p62/SQSTM1 is crucial for transporting damaged proteins from the ubiquitin-proteasome system to the autophagy system [71,72]. However, the autophagy is also dysfunctional in RPE and amplifies the loss of RPE in AMD [70]. Smoking inactivates p62/SQSTM1, decreasing autophagy and reducing the antioxidative system mediated by Nrf-2, resulting in the accumulation of degraded proteins and eventually protein aggregation. Moreover, when p62/SQSTM1, ubiquitin, and other structural proteins are degraded, autophagosomes fuse with lysosomes to form auto-phagolysosomes [71]. The autophagy process is directly linked to both cell survival and programmed cell death, though the specific mechanisms remain unknown. However, in large macular drusen, p62/SQSTM1 levels are elevated, possibly as a compensatory measure for autophagy defects. The p62 protein activates intracellular inflammatory pathways, such as the TNF receptor, triggering the cell death signaling complex and ultimately causing cell death [73].

### 2.9. Inflammation in AMD Pathogenesis

The pathophysiological sequelae of macular drusen include the perpetuation of chronic inflammation and vascular alterations within the retina and choroid. Inflammation, as a response to tissue insult, is a fundamental event in the pathogenesis of AMD, often initiated by drusen [74]. As mentioned earlier, drusen are composite entities containing lipofuscin, amyloid-β, immunoglobulin light chains, factor X, C3, C5b-9 complex, APOE, TIMPs, matrix MMPs, melanin, CRP, vitronectin, fibrinogen, and pentraxins [75]. These deposits, comprising materials originating from RPE cells, along with pigment mottling, are among the earliest clinical indicators of AMD. Retinal and choroidal changes stem from persistent complement activation and inflammation, culminating in the thickening of Bruch’s membrane and compromised permeability, which impairs nutrient transport and waste removal, leading to retinal degeneration and choroidal vascular thinning. Drusen formation is a result of an imbalance between the secretory function of the RPE, which continuously produces large lipoprotein particles, and the age-related transport impairment through the Bruch’s membrane–choriocapillaris complex. The evolution of drusen is intimately linked to RPE viability; drusen accumulation occurs with inefficient RPE clearance and collapses following RPE migration or apoptosis [76,77].

The deterioration of intracellular degradation systems not only increases intracellular protein aggregates and oxidative stress but also triggers the activation of the nucleotide-binding domain, leucine-rich repeat, and Pyrin domain-containing protein 3 (NLRP3) inflammasome. This contributes to AMD pathology through the release of pro-inflammatory cytokine interleukin-1β (IL-1β) and caspase-1-mediated cell death, termed “pyroptosis” [77,78]. The NLRP3 inflammasome is a multiprotein complex that plays a pivotal role in regulating the innate immune system and inflammatory signaling and is activated by oxidative stress, lipids, and impaired autophagy in RPE cells [79,80]. Retinal inflammation is characterized by the activation of microglia, the complement system, and vascular endothelial components. Drusen deposits elicit local inflammation, which in turn plays a significant role in their biogenesis [81]. Systemic inflammation may also contribute to retinal drusen formation in the macula, as suggested by the correlation between complement activation and increased retinal drusen in individuals with intestinal bowel diseases [82].

Understanding these mechanisms provides insight into the complex interplay of oxidative stress, mitochondrial dysfunction, and lipid metabolism in the development of AMD, which is critical for developing targeted therapies [83].

## 3. Imagistic Evaluation of Non-Neovascular AMD

Recent developments in non-invasive imaging technology have markedly improved the capability to visualize and quantify the pathologic features of dry AMD. OCT, FAF, and infrared reflectance (IR) imaging are at the forefront providing detailed images of the macula. OCT has become the new gold standard for the diagnosis of AMD as it provides high-resolution, cross-sectional, and *en face* images of the retina, enabling visualization of its layered structure [84,85,86]. These imaging techniques reveal critical biomarkers of disease progression, such as drusen size and density, GA, and RPE alterations, which are pivotal for staging the disease and prognosis of visual outcomes.

### 3.1. OCT Progression Risk Assessment in Patients with Early and Intermediate AMD

#### 3.1.1. Correlations between OCT Macular Thickness and AMD Changes

Studies have demonstrated a diminution in perifoveal macular thickness with age, as evidenced by OCT, with a notable reduction especially within the inner plexiform layer, outer nuclear layer, and PR and RPE layers [87,88]. In contrast, the foveal thickness, as assessed through the central circle of OCT, does not exhibit age-related changes. Reports have revealed that healthy individuals with genetic susceptibility to AMD, estimated through single nucleotide polymorphism and AMD polygenic risk score, have been associated with reduced macular thickness of the central retinal area, particularly at the level of the outer segment PR and RPE. This may indicate that the PR and RPE thinning of the central retinal subfield is a risk factor for the development of AMD [89,90]. Other longitudinal studies that analyzed the relationship between retinal thickness and the emergence of AMD over a follow-up period of more than 10 years reported that the thinning of the PR layer followed by the RPE and Bruch’s membrane thickening are among the biomarkers for forthcoming AMD development [91].

#### 3.1.2. OCT Evaluation of Drusen

Drusen are hallmark features of AMD and are particularly relevant in the intermediate stage. The identification and monitoring of drusen as an OCT biomarker in intermediate AMD is essential for predicting disease progression, guiding clinical decisions, and evaluating the response to potential therapies. On OCT, they appear as dome-shaped or confluent hyperreflective deposits located beneath the RPE. Drusen are categorized based on their size, number, appearance, and volume. The advantage of OCT evaluation is that it allows for a precise measurement of drusen size, volume, and area. This quantitative analysis is important for monitoring disease progression. An increase in drusen size or number over time can signal a higher risk of progression to advanced stages of AMD, including GA [92,93,94].

Based on their size, drusen can be classified as small, when they are less than 63 microns in diameter; intermediate, between 63 and 125 microns; and large, when they are greater than 125 microns. Small drusen are often seen as normal aging structural changes, while intermediate-size drusen (and not associated with pigmentary abnormalities) are present in the early stages of AMD and may not signify an immediate risk of progression to advanced AMD. Large drusen, on the other hand, are a significant risk factor for progression to severe AMD, including both GA and neovascular AMD [93,95]. In some cases, particularly with larger drusen, there can be a localized detachment of the RPE, termed drusenoid pigment epithelium detachment (PED). This appears on OCT as an area where the RPE is elevated over a homogenous, moderately reflective material, indicating the accumulation of fluid and drusen material beneath the RPE. Another morphometric classification based on both apical height and basal width also includes the category of very large drusen (apical height from 55 to 208 microns and basal width more than 209 microns) [96]. Larger drusen height is correlated with an increased stress on the overlying RPE and PR cells. Predictive OCT biomarkers for GA (especially external limiting membrane (ELM), ellipsoid zone (EZ), and RPE disruption) have been correlated with large drusen height and very large drusen height [97]. SD-OCT studies revealed a correlation between the increased volume, height, and diameters of the drusenoid PED and the rate of drusenoid PED collapse and progression to atrophy. The drusenoid collapse breakpoint was preceded by intraretinal hyperreflective foci and acquired vitelliform lesions [98]. Other studies also reported drusen regression as a risk factor for advancement to GA [99].

The development of sophisticated image processing algorithms has enhanced the accuracy of automatic RPE–drusen complex calculations [100,101,102]. The automated quantification of drusen as well as other retinal abnormalities is useful in monitoring AMD progression. Some SD-OCT devices have integrated quantitative automatic parameters, which can assess changes in the thickness and volume of the RPE–drusen complex and indicate potential advancement to more severe stages of AMD. The Advances RPE Analysis features developed by Zeiss in the Cirrus HD-OCT system provide RPE elevation (indicating drusen detection) and a sub-RPE Slab map (for the evaluation of GA). Also, it analyzes the progression of AMD through parameters like RPE elevation area and volume from a 3 and 5 mm retinal circle centered on the fovea and sub-RPE illumination from a 5 mm retinal circle area. Even though it is a great tool for assessing AMD progression, some studies showed decreased sensitivity and specificity of drusen change quantification measured by Cirrus Advanced RPE Analysis when compared to a multispectral pattern recognition change analysis or expert graders. The multispectral pattern recognition change analysis is a multimodal approach assessment that includes fundus autofluorescence, infrared 815 nm, and green 532 nm ophthalmoscopy image scanning [103].

The multimodal clinical approach provides more accurate and individualized scenarios but comes at the expense of extensive, time-consuming, and cost-ineffective evaluations [94]. The lower reliability of Cirrus Advances RPE Analysis was related to a limited detection of small drusen (the software detects RPE elevations which are greater than 19,4 microns) and to false positive errors (6.1% of normal subjects were identified with RPE elevations) [94,104,105]. The Macustar study report, which used a deep-learning-based algorithm OCT imaging analysis for the determination of RPE–drusen complex volume, found a topographic association between large drusen and hyperreflective foci and incomplete RPE and outer retina atrophy (iRORA) lesions in patients with iAMD [106].

Drusen display varied reflectivity patterns on OCT. They can be broadly categorized into high, medium, and low reflectivity, depending on their composition. High-reflective drusen usually indicate a more solid composition. Low-reflective drusen may suggest a softer, more fluid-like consistency. Most often, drusen have medium or low internal reflectivity and do not exhibit depolarizing material on polarization-sensitive OCT. The reflectivity of the drusen is reported in relation to the PR layer [107,108]. Studies reported that drusen with low reflectivity and RPE and EZ overlaying damage have a higher progression rate compared to high-reflectivity drusen [109]. Other reflectivity traits which have been associated with progression towards geographic atrophy are heterogenous internal reflectivity of the drusen and hyporeflective cores [110,111,112,113]. The distribution of drusen has also been associated with different core reflectivity, the peripheral ones having a higher reflective core compared to the central drusen [108]. Calcified drusen have been shown to display on OCT B-scans hyporeflective internal cores, within heterogenous internal reflectivity and a hyperreflective top. For the convenient visualization and monitoring of the calcified drusen, the *en face* mode of the OCT can also be used (they are seen as hypotransmission defects) [114]. Calcified drusen are predictive of the development of RPE and PR atrophy and eventually GA [114,115].

While conventional OCT remains a well-established tool for the characterization of drusen, additional imaging techniques like Polarization Sensitive Optical Coherence Tomography (PS-OCT) may offer supplementary information. PS-OCT provides a unique and advanced imaging capability to visualize and characterize drusen in patients with AMD, offering insights that are not readily available with conventional OCT imaging [116]. PS-OCT exploits the polarization properties of light to enhance tissue contrast and provide detailed information on the microstructure of the retina. By measuring changes in the polarization state of light reflected from the retina, PS-OCT can provide enhanced contrast of drusen against the surrounding tissue. This is particularly useful for identifying small or early-stage drusen that might not be as apparent on intensity-based OCT scans [107]. PS-OCT can also provide information on the birefringent properties of drusen, which may offer insights into their composition and structure. Since drusen can vary in their content, including lipids, proteins, and minerals, PS-OCT may help in distinguishing between different types of drusen based on their polarization characteristics [107,117].

#### 3.1.3. Other OCT Biomarkers Related to AMD Progression to Advanced Stages

1. Hyperreflective foci overlying drusen

**Figure 1:**
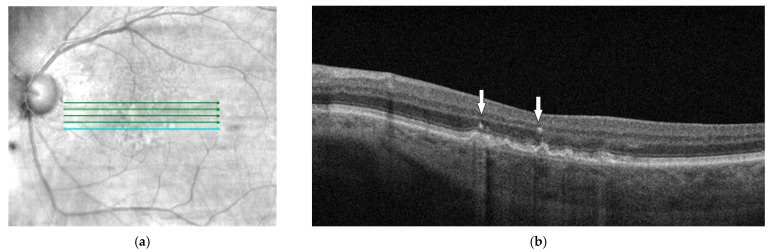
(**a**) Red-free fundus photography of macula with large drusen; (**b**) OCT B scan corresponding to the blue line from image (**a**) that shows hyperreflective foci which are localized adjacent to the drusen apex (arrow).

Hyperreflextive foci (HRF) are defined, well-circumscribed, usually round lesions that are characterized by high OCT reflectivity which is similar to that of RPE (Figure 1). They are localized on SD-OCT in the inner neuro-sensory retina or adjacent to the drusen apex. On eye fundus examination, they are generally detected as hyperpigmentations. Nevertheless, a recent study has demonstrated that actually only two-thirds of the intraretinal HRF present on OCT correspond to hyperpigmentations seen on the color fundus photography (CFP) [118]. HRF are widely considered displaced RPE cells, outer segment PR debris, or pigment granules [99]. Recent studies showed that the majority of HRF are melanosome mononuclear phagocytes, which are named melanophages. These are a type of microglial cell which has been shown to play a critical role in PR degeneration, as well as in other age-related retinal diseases [119]. Other studies have linked the presence of HRF on OCT to transdifferentiated RPE, which has increased immunoreactivity for immune markers but decreased immunoreactivity for retinoid markers [120].

Their presence is of high clinical importance as they are considered predictive OCT biomarkers for progression to both neovascular AMD and GA [119,121,122]. In patients with intermediate AMD and HRF, the mean time for advanced AMD onset was 29 ± 12 months [121]. An increased number of HRF is associated with more severe stages of AMD. Correlations between structural changes and functional abnormalities have identified that eyes with HRF exhibit significantly longer dark adaptation periods, local reductions in visual sensitivity, and low-luminance visual acuity [123,124,125]. Similar features to HRF are seen in hyperreflective specks which are comparably smaller in dimeter, have lower reflectivity than the PRE layer, and are localized in the Henle fiber and ONL layers on OCT. They represent lipofuscin granules which have migrated from the PR layer towards a more inner layer of the retina [125,126]. On functional evaluation, hyperreflective specks are associated with decreased contrast sensitivity, low-light level deficit, and decreased mesopic and scotopic light sensitivity [125].

The localization of HRF may be a prediction factor for AMD progression. Parafoveal localization of HRF was associated with atrophic macular lesions, while foveal distribution and co-localization of drusen beneath the HRF were linked to the growth of macular neovascularization [126]. Also, in eyes with intermediate AMD, the presence of HRF overlying the drusen has been associated with a decreased choriocapillaris flow when compared to that beneath drusen without HRF [127].

2. Ellipsoid zone disruption

EZ refers to the junctions between the outer segment and the inner segment of the PR cells. The disruption of EZ is another OCT parameter which has been linked to the progression of AMD to advanced stages [11,64,99]. There is 2.5-fold risk increase of developing advanced stages of AMD in patients that present with EZ disruption compared to those without it [128]. EZ changes have been noticed as early as 4 years before GA onset [85]. In a multivariate analysis model, some results even suggest that EZ disruption together with iRORA are among the best progression predictor biomarkers. When both OCT abnormalities are present, there is a high risk of advancement to cRORA within the next two years [128]. EZ disruption has been recounted as the precursor for the succeeding RPE atrophy in patients with GA [1]. The pattern of EZ disruption has been described as a possible indicator for GA pattern progression. Nevertheless, it was not a reliable quantitative parameter for the prediction of GA area at 1 year [86]. Other reports that analyzed the relationship between drusen and other biomarkers of atrophy present on OCT revealed an increasing frequency of EZ disruption with greater drusen height. EZ disruption was present in 17.9% of subjects with small drusen, 46.2% of those with intermediate drusen, and 86.8% of those with large drusen [97]. In early forms of non-vascular AMD, OCT studies showed that the absolute reflectivity of the EZ was significantly lower in patients with AMD compared to healthy controls. Also, when the relative reflectivity of the EZ (EZ to external limiting membrane reflectivity) values were compared between controls and AMD groups, the latter showed decreased values [129].

Functional assessments that applied microperimetry testing in patients with early AMD showed a decreased differential light sensitivity in those with associated EZ disruption despite retaining an adequate visual acuity. The reduction in differential light sensitivity was related to the decreased thickness of the PR outer segment and increased thickness of the RPE [130,131]. Nevertheless, not all studies have found an association between the retinal sensitivity and integrity of EZ. They reported only a correlation between fixation stability abnormalities on microperimetry and inner EZ disruption [132]. Other reports also revealed functional abnormalities, and the presence of EZ disruption was consistently associated with delayed rod-intercept time and impaired dark-adaptation [133]. On CFP, hyperpigmented lesions that were not associated with hyperreflective foci on OCT were demonstrated in 86% of cases of EZ disruptions [134].

3. Subretinal drusenoid deposits (reticular pseudodrusen)

SDDs, also described by some studies as reticular pseudodrusen, represent yellowish (on CFP) deposits of granular, hyperreflective material (on OCT) localized in the subretinal space, interlaced between the RPE layer and the PR cells [99,135]. Their prevalence increases with age and is more common in females. Even though they appear also in other retinal diseases, their highest association is with AMD. Evidence suggests a high correlation between the presence of SDDs and the progression to advanced stages of AMG, especially GA but also the neovascular subtype [135,136]. Reports also suggest a possible hereditary aspect of SDDs in first-degree relatives [137]. Furthermore, even though multiple studies have not found a strong correlation between AMD and cardio-vascular diseases, the presence of SDDs in this specific phenotype of AMD patients appears to emerge as an independent risk factor for systemic vascular diseases including stroke [138,139]. While SDDs are not very accurately detected on regular clinical examination or color fundus photography, they are best visualized on SD-OCT, infrared reflectance, or multimodal imaging [140,141]. SDD lesions undergo a certain lifecycle characterized by growth, expansion in the EZ, and the final stage of regression associated with perturbations of the PR and RPE. These correspond to stages 1–3 on OCT. On adaptive optics scanning laser ophthalmoscopy examination, SDDs corresponding to stage 1 on OCT are associated with decreased reflectivity of the overlying PR, those corresponding to stage 2 are associated with significantly reduced PR reflectivity, while those corresponding to stage 3 are associated with a hyporeflective annular zone of degenerated PR [142]. Some studies nevertheless reveal that a small proportion of the SDD lesions reappear after the third stage of regression [143]. Moreover, the presence of SDDs was associated with decreased choroidal vascularity index on foveal horizontal OCT scans. The choroidal vascularity index was even more reduced in early AMD patients with SDDs compared to those with conventional drusen [144].

As SDDs imply abnormalities related to rods and first appear in the perifoveal area, they are associated with decreased retinal sensitivity and dark-adaptation evident by prolonged rod-intercept time. Mesopic and scotopic sensitivity were significantly decreased in AMD patients that were associated with predominantly SDDs when compared to those with drusen and without SDDs [145,146,147].

4. Choroidal hyper-transmission

Choroidal hyper-transmission defects on OCT in the context of AMD refer to specific findings associated with atrophy. Choroidal hyper-transmission defects appear as areas where the OCT light penetrates more deeply into the choroid than normal. This results in increased brightness or “hypertransmission” of the OCT signal below the retina. These areas may appear as more “lucid” zones compared to the surrounding tissue due to thinning or atrophy of the overlying RPE and the PR layer. The RPE normally blocks or scatters the passage of light, so its thinning or absence allows for an increased signal into the choroid, causing the hypertransmission effect. One of the robust ways to display the hypertransmission defects is on the *en face* feature, on the sub-RPE slab of the OCT [99]. One of the advantages of this setting is that it allows for the measurement and quantification of atrophy enlargement. Persistent hypertransmission defects that have the greatest linear dimension over 250 microns on *en face* OCT imaging have been strongly proposed as a risk factor for the progression towards GA [148,149]. Some studies have proposed this technique as a method that offers new endpoints for clinical trials aimed at evaluating treatments that could decelerate the progression from iAMD to late-stage AMD [150]. Nevertheless, some reports still consider FAF a more sensitive diagnostic method for the detection of RPE atrophic changes, when compared to SD-OCT. These results were evident in instances of iAMD where early atrophic lesions overlying the drusen are not concurrently identified in FAF and SD-OCT. For some particular cases, a multimodal approach could be the most appropriate method for evaluation [151].

5. iRORA

**Figure 2:**
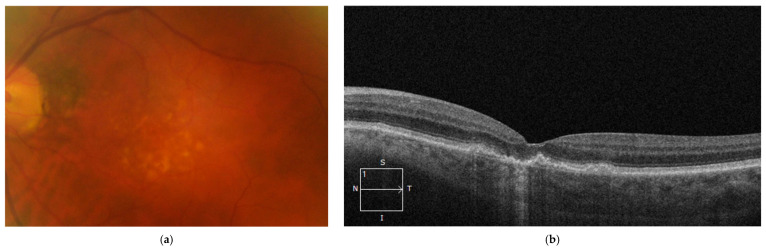
(**a**) CFP of macula with large drusen; (**b**) OCT B scan that shows iRORA lesion, note loss of ellipsoid zone, ELM, attenuation of RPE, and hypertransmission signal into the choroid (<250 microm).

The Classification of Atrophy Meeting (CAM) group has classified the pre-GA lesions or atrophic lesions based on OCT parameters. While GA is a term which is related to the atrophy of the outer retina seen on CFP as an area of hypopigmentation and an exposure of choroidal vasculature, recent recommendations suggest the use of the new OCT nomenclature iRORA and cRORA for the evaluation of atrophic lesions associated with non-neovascular AMD [152]. The CAM group has highlighted the importance of AMD atrophic lesions’ evaluation in accordance with the anatomical retinal layer which has been affected. iRORA represents an incomplete retinal pigment epithelial and outer retinal atrophy (Figure 2), while cRORA represents a complete retinal pigment epithelial and outer retinal atrophy of at least 250 microns on a horizontal OCT B scan. iRORA lesions should not be used in the presence of a retinal tear. Also, for the diagnosis of iRORA, the following criteria must be met on OCT: hypertransmission into the choroid, disruption or attenuation of RPE, and signs of deterioration of the PR layer which is overlying the RPE [152]. In general, standard OCT is used for the identification of iRORA lesions; nevertheless, high-resolution OCT has been superior in the detection and classification of the atrophic lesions, enhancing the assessment reliability. iRORA is a noteworthy OCT biomarker as these lesions are predecessors for GA and a high-risk progression factor to the advanced stages of AMD [128]. Another relevant aspect to note is that the term “nascent GA” can be used when iRORA lesions are present but there is no association with previous or present signs of macular neovascularization [152].

Subjects with iAMD have a higher chance to develop iRORA lesions if they have other lesions like drusen ooze on OCT, an associated decreased microperimetric retinal sensitivity, or increased inner choroid flow deficits [153,154]. The prevalence of iRORA lesions is between 10% and 33% in subjects with intermediate AMD [106,155]. In patients who already have GA, iRORA lesions which were present around the GA area progressed to cRORA in a high percentage of cases (81.8% at 12 months) [156]. Also, studies have shown an accelerated conversion of iRORA lesions to cRORA, in subjects which associated GA in the fellow eye. In these cases, the mean time interval was 7 months between the two stages of retinal atrophy [157]. For bilateral cases of AMD, the timeframe for the emergence of iRORA is around 3.5 years between the two eyes [158]. Nevertheless, complement inhibitors, like pegcetacoplan, emerge as a new therapeutic option, lowering the progression rates of iRORA to cRORA [156].

### 3.2. OCT Evaluation of Geographic Atrophy

GA is characterized as a distinct, typically round or oval region of reduced or absent pigmentation, allowing for greater visibility of the choroidal vessels beneath the retina. This region must be at least 175 μm in diameter on color fundus photography images. Given the long-standing use of the term GA in academic texts over the years, the CAM group has recommended maintaining the term specifically for cases of atrophy where choroidal neovascularization is absent, either currently or as determined from CFP.

**Figure 3:**
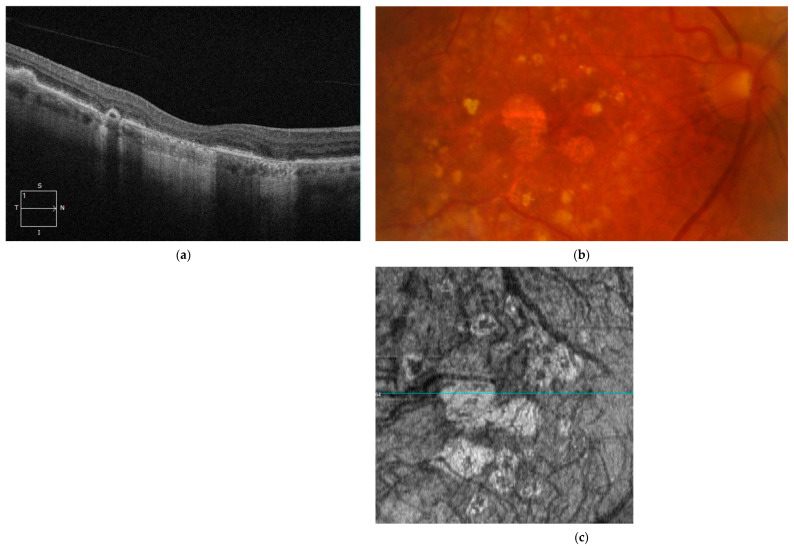
(**a**) Complete RPE and outer retinal atrophy (cRORA) and presence of choroidal hypertransmission; (**b**) CFP showing patches of geographic atrophy; (**c**) hypertransmission defect highlighted on *en face* OCT (blue color-coded line is corresponding to the B scan shown in (**a**)).

Therefore, GA is considered a specific instance within the broader category of complete retinal pigment epithelium and outer retinal atrophy (cRORA), which includes macular atrophy both in the presence and absence of CNV (Figure 3). As mentioned in the previous point, cRORA is a term used to define RPE and outer retinal atrophy, and choroidal hypertransmission of at least 250 microns in diameter, on OCT findings. The term “nascent GA” should be preserved to describe incipient iRORA where choroidal neovascularization is not observed on OCT [152,159].

OCT studies that evaluated the progression rate of GA in AMD patients showed a mean rate of GA progression of 1.49 mm2/year. This value is quite similar and only marginally lower than the GA progression rate measured through color fundus photography and fundus autofluorescence (1.49–1.99 mm^2^/year) [160,161]. Generally, most reports show an adequate correspondence between the area of atrophy measured on FAF or CPF and the measurements of cRORA on OCT [160,162,163,164]. When comparing mean atrophy progression rates using cRORA criteria versus hypertransmission criteria on SD-OCT, the latter showed slightly lower values [164]. Also, the focality of the GA at baseline is an important factor that impacts the progression rate, with multifocal lesions having a higher progression rate [160]. From the multifocal cRORA lesions, the “diffuse trickling” patterns have been shown to have an accelerated progression of atrophy [163]. Female sex is another risk factor for GA advancement [160].

### 3.3. Particular Features Regarding OCT Biomarkers in Patients with GA That Are Candidates for Complement Inhibitor Therapy

In recent years, significant breakthrough therapies have emerged for the treatment of GA. Pegcetacoplan is an important therapeutic candidate in the treatment of GA and the first FDA-approved intravitreal injection drug for this disease. It functions as a complement inhibitor that specifically targets the C3 protein in the complement pathway, a part of the immune system implicated in the pathogenesis of AMD. By inhibiting C3, pegcetacoplan aims to slow down or halt the progression of GA by reducing the inflammatory and immune-mediated damage to the retinal cells. Although recent therapeutic developments are very exciting, the identification of appropriate biomarkers for the evaluation of progression rates will be crucial for proper assessment. While we have already described in the upper sections of this paper the OCT biomarkers that are related to retinal atrophy progression, we would like to emphasize some particular features of OCT evaluation in patients that are candidates for complement inhibitor therapy. Recent studies have revealed different behaviors of the progression rates of the recently developed cRORA lesions compared to advanced lesions, with the latter having a larger growth rate [165,166]. This is a meaningful aspect when following patients under treatment. OCT segmentation analysis shows a significantly reduced atrophy enlargement rates for RPE and ELM layers in early cRORA lesions compared to advanced ones. In late cRORA lesions, the enlargement rate of the RPE, PR, and ELM layers were comparable [1]. Also, another significant finding for the evaluation of GA therapies is that the PR loss is consistently greater than that of the RPE. The PR layer atrophy extends past the edges of the cRORA lesions and is an early indicator of impending RPE atrophy in GA. Patients with early cRORA lesions have greater PR/RPE loss ratios compared to those with more progressed cRORA lesions. As the disease naturally progresses, the RPE loss to EZ loss ratio shrinks as RPE degeneration catches up [165,166,167]. Moreover, for the evaluation of the therapeutic response, assessment of the GA lesion topography becomes useful. Lesions that do not involve the fovea have a higher progression rate than those that do include the fovea. In these cases, the treatment with complement inhibitors becomes particularly beneficial [161,168,169].

### 3.4. Directional Optical Coherence Tomography in AMD

Directional Optical Coherence Tomography (dOCT) is a variation of the conventional OCT technology that provides additional insights by varying the incident beam’s angle to highlight and differentiate structures based on their directional reflectivity. dOCT uses multiple beam light positions which are displaced at the level of the pupil entrance. This approach enhances the contrast of retinal images and reveals more details about the retina’s microstructure [170]. In the context of AMD, dOCT can provide additional information related to the early stages of the disease or to AMD progression. Investigations have disclosed that in dry AMD patients, the variation in the EZ reflectivity by the incident angle of light was less than that of the controls for both AMD segments between drusen as well as for the retina overlying the drusen. This finding implies potential photoreceptor orientation or structural integrity disruptions [171]. Moreover, dOCT has been recognized for its superior capacity in delineating PR visibility in regions overlying drusen, surpassing the clarity achieved with adaptive optics imaging modalities [172]. Additionally, alterations in the outer nuclear layer, indicative of PR loss in retinal degeneration, are more distinctly captured by dOCT, whereas traditional OCT techniques exhibit limitations in demarcating Henle’s fiber layer from the outer nuclear layer [173]. Furthermore, dOCT is a powerful tool to assess the RPE layer. Reports have indicated a correlation between a lower variability in directional scattering recorded by dOCT and an increase in the melanin concentrations found in the RPE. Studies performed in mice with varying melanin levels found that the intensity of light backscattered varies with the angle of illumination, especially in melanin-free layers, while it is less sensitive in the inner/outer segment junctions [174]. In the context of Abca4−/− mice, a genetic model of Stargardt disease, dOCT has been validated as an efficacious method for assessing the decrement of RPE melanosomes [175]. The presented data highlight the utility of dOCT in the risk assessment of AMD patients, considering the depletion of RPE melanin as a potential risk factor for AMD-related pathological alterations.

### 3.5. OCT-Angiography Changes in Early and Intermediate Non-Neovascular AMD

**Figure 4:**
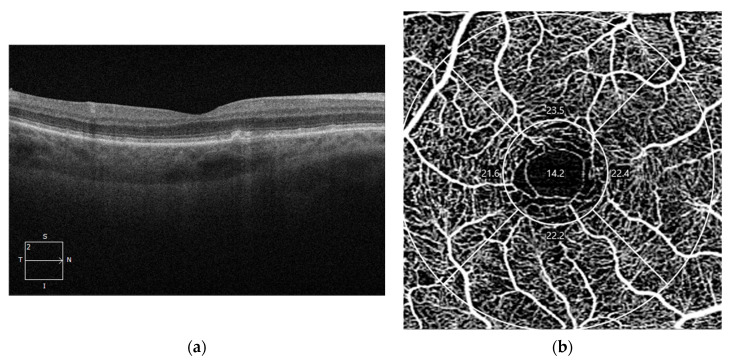

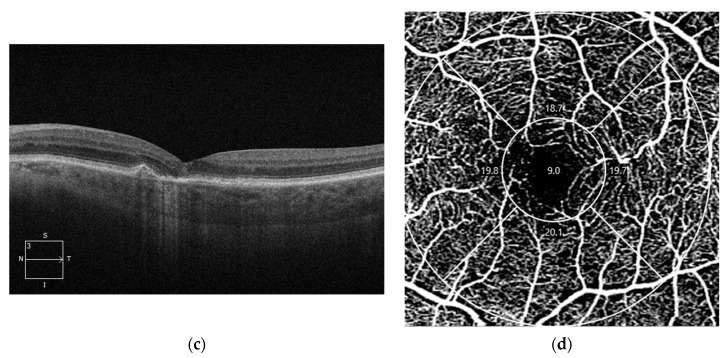
OCT and OCTA of a patient with non-neovascular AMD. (**a**) OCT scan of the right eye showing an early stage of AMD characterized by the presence of drusen seen as elevations of the RPE hyperreflective layer; (**b**) OCTA of the right eye showing normal vessel density of the superficial retinal plexus; (**c**) OCT scan of the left eye of the same patient showing a more advanced form of non-neovascular AMD characterized by iRORA lesions (RPE disruption and outer retina atrophy); (**d**) OCTA of the left eye displaying reduced vessel density in the superficial vascular plexus of the retina.

OCTA provides three-dimensional images of retinal and choroidal vascularization, allowing for the correlation of vascular changes with structural alterations seen on OCT [176]. In normal eyes, OCTA of the choriocapillaris reveals a dense, homogeneous network with a fine pattern at the macular level. Early and intermediate dry age-related macular degeneration is characterized by drusen and pigmentary abnormalities, with significant changes in retinal vascularization as the stage of the disease progresses [177]. OCTA in early dry AMD shows a generalized reduction in choriocapillaris density compared to normal individuals, with focal areas of choriocapillaris loss appearing as dark zones. These areas may be accompanied by a displacement of larger choroidal vessels into the space previously occupied by the choriocapillaris, with these changes becoming more pronounced in advanced stages [178]. In intermediate dry age-related macular degeneration, OCTA analysis reveals an impairment of the choriocapillaris, particularly beneath and in the vicinity of drusen, but also a reduction in the vessel density of the superficial and deep retinal plexus (Figure 4) [177,179]. Patients with reticular pseudodrusen exhibit specific alterations in the choriocapillaris, including a decrease in choroidal volume and thickness, an elevated choroidal vascularity index, and increased choroidal intensity [180]. Choriocapillaris impairment on OCTA can serve as a predictor for disease progression, where decreased choriocapillaris flow may predict the growth of pre-existing drusen and the risk of developing new drusen [181]. The reduction in choriocapillaris flow is more severe in patients with hyperreflective foci, especially beneath these hyperreflective lesions. Considering that these foci correlate with progression to late-stage AMD and the onset of GA, it can be considered that choriocapillaris flow impairment itself may act as a predictor of disease progression [127].

### 3.6. OCT-Angiography Changes in Geographic Atrophy

**Figure 5:**
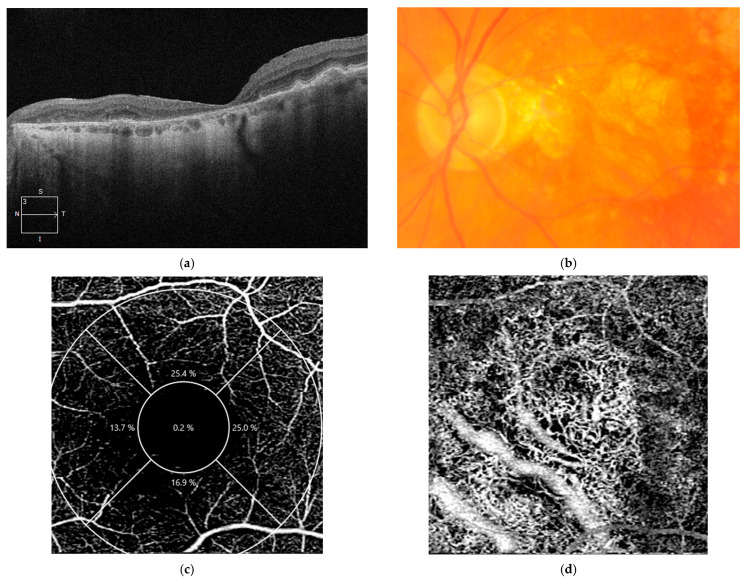
OCT, CFP, and OCTA from a patient with advanced non-neovascular AMD and geographic atrophy. (**a**) Horizontal OCT scan recorded at the level of the fovea showing atrophy of the RPE, PR, ELM, and outer nuclear layer; (**b**) CFP showing geographic atrophy marked by a delineated area of hypopigmentation in the macular zone and visualization of the underlying choroidal vascularization; (**c**) OCTA scan showing a significant reduction in the blood perfusion in the superficial vascular complex (percentages represent perfusion densities from a 3 × 3 mm scan); (**d**) outer retina to choriocapillaris OCTA scan displaying the loss of the choriocapillaris which enables the underlying larger choroidal vessels to become visible.

Even though GA is primarily assessed through CFP and FAF, which serve as the gold standard for evaluating its progression, OCTA can also provide complementary information (Figure 5) [182,183]. OCTA shows a decreased flow in the choriocapillaris within atrophic areas and provides a more detailed visualization of choroidal vessels [178]. Therefore, GA manifests as a loss of the choriocapillaris complex at the level of retinal pigment epithelium atrophy [178]. As the choriocapillaris is lost, there is regression of the middle portions of the choroid and the deeper larger choroidal vessels ascend to the level of the inner choroid [184]. The choriocapillaris is also impaired in the peripheral macula in patients with GA, compared to normal individuals or those with choroidal neovascularization [185]. The most significant impairment of the choriocapillaris on OCTA is in the immediate vicinity of GA and serves as a predictive factor for the extension of the GA area [186]. In light of these considerations, OCTA has demonstrated that the impairment of the choriocapillaris complex should be regarded as a risk factor for the progression of GA. It needs to be evaluated to determine the effectiveness of new therapeutic methods aiming to slow down the progression of GA.

### 3.7. Fundus Autofluorescence Imaging in Early and Intermediate AMD

Even though OCT is the new standard for the evaluation of structural changes related to early and intermediate AMD, FAF remains an important imaging modality which completes the diagnosis. On autofluorescence, drusen can exhibit a variable hyperautofluorescent appearance due to the presence of lipofuscin in the drusen or in the overlying RPE. Alternatively, they may appear hypoautofluorescent due to degeneration of the RPE cells or regression of the drusen [187]. Small- and medium-sized drusen often have a variable autofluorescent appearance and may go unnoticed. While soft drusen appear hyperautofluorescent, particularly in the periphery compared to the center, cuticular drusen have hypoautofluorescent features [187]. On the other hand, drusenoid detachments of the RPE present with a mottled pattern of hypo- and hyperautofluorescence [188].

Reticular pseudodrusen manifest as round or oval-shaped, small (with diameters between 50 and 400 microns, usually under 200), homogeneous, clustered hypofluorescent areas, arranged regularly in a network, and are predominantly localized in the upper part of the fovea [187]. They are believed to appear hypoautofluorescent due to their subretinal location, which obstructs the reflectivity of lipofuscin in the RPE [188]. Considering that the presence of these reticular drusen in early and intermediate age-related macular degeneration is associated with an increased risk of progression to late-stage disease, these patients require a more frequent follow-up [189,190].

The autofluorescence changes in cases of early AMD have been classified according to The International Fundus Autofluorescence Classification Group-IFAG as eight distinct autofluorescence patterns: normal, minimal change, focal increase, patchy (features at least one large area > 200 microns in diameter of increased FAF, with indistinct borders), linear (features at least one linear area of increased FAF with well-defined borders), lace-like, reticular (features multiple, round, small, < 200 microns, regularly networked areas of decreased FAF), and speckled [191]. In intermediate AMD, the most commonly encountered changes involve punctate areas of increased autofluorescence. Additionally, punctate areas of decreased autofluorescence and linear areas of increased autofluorescence can also be observed [192].

### 3.8. Fundus Autofluorescence Imaging in Geographic Atrophy

FAF imaging is pivotal in detecting and delineating GA. In the late-stage AMD form, areas of atrophy show decreased autofluorescence due to the loss of RPE cells, appearing as well-defined hypoautofluorescent zones against a background of variable autofluorescence. FAF is highly sensitive for the early detection of GA and is invaluable in monitoring the enlargement and progression of atrophic areas over time. On autofluorescence, GA can present as a well-demarcated unifocal or multifocal region of hypoautofluorescence [192]. GA often develops in the central or parafoveal macular region and may progress towards the peripapillary area. The areas of GA in the parafoveal region typically merge into a ring-like or horseshoe pattern, eventually affecting the initially spared central region [187]. The new lobules may exhibit a lower intensity of hypoautofluorescence, appearing more gray than dark as a result [193]. Surrounding the GA, there may be areas of irregularly shaped punctate or large hyperautofluorescence, suggesting that cellular death is likely occurring in these zones [194]. FAF is particularly capable of detecting small or discrete areas of GA compared to other imaging methods, making it especially useful for GA progression assessment [193,195].

### 3.9. Color Fundus Photography

While less sensitive than FAF and OCT, color fundus photography remains a standard tool for documenting the appearance of retina lesions. It provides a broad overview of the retinal condition, including the presence of drusen, pigmentary changes, and atrophic patches [196,197]. While sensitivity values for atrophic lesions are quite high, early features of AMD like reticular drusen or areas of hypopigmentation can be detected on CFP with lower sensitivity [5].

## 4. Conclusions

In conclusion, the imaging evaluation of geographic atrophy in patients with non-neovascular AMD is multifaceted, integrating various technologies to provide a comprehensive understanding of the disease. These imaging modalities not only aid in the accurate diagnosis and monitoring of AMD but also contribute to the understanding of its pathophysiology, which is essential for the development of future therapeutic options. As imaging technology evolves, it is expected that our ability to detect, monitor, and potentially treat AMD, especially GA, will improve, offering hope for patients affected by this currently irreversible condition.

## Data Availability

Not applicable.
